# Impact of Thermal Degradation of Cyanidin-3-*O*-Glucoside of Haskap Berry on Cytotoxicity of Hepatocellular Carcinoma HepG2 and Breast Cancer MDA-MB-231 Cells

**DOI:** 10.3390/antiox7020024

**Published:** 2018-01-27

**Authors:** Eric Pace, Yuanyuan Jiang, Amy Clemens, Tennille Crossman, H.P. Vasantha Rupasinghe

**Affiliations:** 1 Department of Plant, Food, and Environmental Sciences, Faculty of Agriculture, Dalhousie University, Truro, NS B2N 5E3, Canada; pace.eric.arthur@gmail.com (E.P.); yyjiang@dal.ca (Y.J.); am661344@dal.ca (A.C.); tennille.crossman@dal.ca (T.C.); 2 College of Science, Sichuan Agricultural University, Yaan 625014, China

**Keywords:** anthocyanin, food processing, antioxidant, cancer, cell viability, phenolic compounds

## Abstract

Cyanidin-3*-O*-glucoside (C3G), the predominant anthocyanin in haskap berries (*Lonicera caerulea* L.), possesses antioxidant and many other biological activities. This study investigated the impact of temperature and pH on the degradation of the C3G-rich haskap fraction. The effect of the thermal degradation products on the viability of hepatocellular carcinoma HepG2 and breast cancer MDA-MB-231 cells was also studied in vitro. Using column chromatography, the C3G-rich fraction was isolated from acetone extracts of haskap berries. The C3G stability in these fractions was studied under elevated temperatures (70 °C and 90 °C) at three different pH values (2.5, 4, and 7) by monitoring the concentration of C3G and its major degradation products, protocatechuic acid (PCA) and phloroglucinaldehyde (PGA), using liquid chromatography mass spectrometry. Significant degradation of C3G was observed at elevated temperatures and at neutral pH. Conversely, the PCA and PGA concentration increased at higher pH and temperature. Similar to C3G, neutral pH also has a prominent effect on the degradation of PGA, which is further accelerated by heating. The C3G-rich fraction exhibited dose-dependent inhibitory effects on cell metabolic activity when the HepG2 cells were exposed for 48 h. Interestingly, PGA but not PCA exhibited cytotoxic effects against both MDA-MB-231 and HepG2 cells. The results suggest that thermal food processing of haskap could influence its biological properties due to the degradation of C3G.

## 1. Introduction

Haskap (*Lonicera caerulea* L.) berries, also known as blue honeysuckle and honeyberry, are a relatively new crop in North America. Berries of this genus have been used for medicinal purposes in Russia and some Asian countries for thousands of years [1,2]. There are three major cultivars of this berry being produced in Canada, and they have attracted interest due to their relatively high concentration of health-promoting anthocyanins when compared with other commonly consumed fruit [1,3,4]. Many studies have indicated that anthocyanins have the potential to be effective in cancer chemoprevention. Anti-proliferative effects on multiple cancer cell types in vitro, pro-apoptotic effects on cancer cell lines, reduction in inflammatory pathway expression, anti-angiogenesis effects, and induced differentiation have been observed [5,6,7,8,9,10,11,12]. Anthocyanins administered in vivo have reduced cancer development in animals treated with carcinogens, and in animals with the common hereditary development of colon, lung, skin and esophageal cancers [13,14,15]. Anthocyanins, which degrade rapidly, are the least stable class of flavonoids. The stability of anthocyanins is affected by pH, light exposure, oxidation, enzymatic action, and the presence of metal ions [16]. Under acidic conditions (pH < 3), anthocyanins exist in their most stable form, red flavylium cations. When the pH is between four and five, anthocyanins undergo hydroxylation and form a colorless pseudo base. At a higher pH (pH > 6), anthocyanins exist as a quinoidal base and exhibit a blue color. Under alkaline conditions, anthocyanins can also form chalcones, which exhibit a yellow color, and can degrade to produce phenolic acids [17].

Cyanidin-3-*O*-glucoside (C3G), which is the predominant anthocyanin in haskap berries [18,19], thermally degrades into protocatechuic acid (PCA) and phloroglucinaldehyde (PGA) [20]. PCA has exhibited anti-inflammatory and anti-oxidative effects and has reduced liver toxicity in vivo [21]. PCA may also possess anti-tumoral properties, inducing apoptosis in human leukemia cells, as well as in human salivary gland carcinoma (HSG1) cells [22]. Conversely, PCA has also been shown to increase proliferation in 12-*O*-tetradecanoylphorbol-13-acetate (TPA)-induced mouse skin tumors [23]. Currently, limited studies are showing the antiproliferative effects of PGA, with only two studies suggesting anti-proliferative effects against colon cancer cells Caco-2 [24,25]. Recent studies investigating the pharmacokinetics of C3G in humans have identified the presence of both PCA and PGA in the blood serum of individuals who have ingested labeled C3G [26,27]. Therefore, this study aims to characterize C3G degradation, PCA and PGA formation in the haskap extract, and the antiproliferative properties of C3G and its major degradation products against hepatocellular carcinoma HepG2 and breast adenocarcinoma MDA-MB-231 cells.

## 2. Materials and Methods

### 2.1. Plant Material

Haskap berries of the Tundra cultivar (TN) were obtained from LaHave Natural Farm, Blockhouse, Nova Scotia, Canada and stored at −20 °C.

### 2.2. Chemicals and Reagents

High performance liquid chromatography (HPLC) grade methanol, ethyl acetate, 88% formic acid, acetone, phosphate buffered saline (PBS), dimethyl sulfoxide (DMSO), Eagle Minimum Essential Growth Medium (EMEM), Dulbecco’s Modified Eagle’s Medium (DMEM), penicillin-streptomycin, fetal bovine serum (FBS), PCA, PGA, doxorubicin (DOX), and all other chemical reagents were purchased from Sigma-Aldrich (Oakville, ON, Canada). C3G was purchased from Extrasynthese (Genay Cedrix, France). Trisodium citrate and anhydrous dibasic sodium phosphate were purchased from Fisher Scientific (Ottawa, ON, Canada). Sorafenib was purchased from Cayman Chemicals (Cedarlane, Burlington, ON, Canada). 5-[3-(Carboxymethoxy) phenyl]-3-(4,5-dimethyl-2-thiazolyl)-2-(4-sulfophenyl)-2H-tetrazolium inner salt (MTS) was purchased from Promega (Madison, WI, USA).

### 2.3. Preparation of Extracts

#### 2.3.1. Preparation of Crude Extract

The crude extract (CE) was prepared using frozen haskap berries (500 g) extracted in semi-dark conditions. Berries were ground in a blender (Model HBB909, Hamilton Beach Brands Inc., Glen Allen, VA, USA) in a solution of 70:28:2 acetone:deionized water (DI H_2_O):formic acid. For every 1 g of frozen berries, 4 mL of solvent was used. The blended berries and solvent were filtered through six layers of cheese cloth, then vacuum filtered through Whatman # 8 filter paper (Fisher Scientific, Ottawa, ON, Canada). Samples were subjected to rotary evaporation to remove the solvent, then frozen at −80 °C and freeze-dried. Crude extracts were then stored at −80 °C.

#### 2.3.2. Preparation of C3G-Rich Fraction

C3G-rich fraction (PE) was prepared in the same manner as crude extracts with additional steps, as described in a previous method [28]. Following rotary evaporation to remove acetone, the resultant liquid was added to equal volumes of ethyl acetate in a separatory funnel under dark conditions and was allowed to separate for 12 h before the aqueous phase was collected. Trace amounts of ethyl acetate were removed from the aqueous phase via rotary evaporation. A column (3.8 × 45 cm, Sati International Scientific Inc., Dorval, QC, Canada) was packed with 600 g adsorbent (Sorbent SP207-05 Sepabeads Resin Brominated Styrenic Adsorbent: particle size 250 µm, surface area 630 m^3^/g, Sorbent Technologies, Atlanta, GA, USA) and conditioned with methanol followed by DI H_2_O acidified to pH 2.5 with hydrochloric acid (HCl). The crude extract was applied to the column and washed with 1 L DI H_2_O acidified to pH 2.5 with HCl. C3G-rich fraction was eluted with about 500 mL of methanol. Methanol was removed using rotary evaporation and then freeze-dried to prepare dried PE, which was stored at −80 °C.

### 2.4. Degradation Kinetics

#### 2.4.1. Sample Preparation

PE was prepared at a concentration of 40 mg/mL in a citrate–phosphate buffer (0.2 M tri-sodium citrate, 0.3 M dibasic sodium phosphate). Portions of this solution were acidified to pH 2.5, 4, and 7 with citric acid and stored in 2-mL aliquots in 2-mL sealed amber glass vials. Samples were incubated in triplicate, in a water bath at either 70 °C or 90 °C, for periods of 0, 0.5, 1, 2, 4, or 8 h. After incubation, samples were immersed in an ice bath to limit further degradation. Samples were then stored at −80 °C until analysis. Pure PGA solutions (50 μg/mL, 2 mL) were incubated in a 90 °C water bath for 0, 0.5, 1, 2, 4, and 8 h periods in 2-mL sealed amber glass vials at pH of 2.5, 4, and 7. Each of the heated solutions was diluted with 2 mL MeOH and filtered before HPLC analysis.

#### 2.4.2. Quantification of Total Monomeric Anthocyanins, C3G, PCA, and PGA

Total monomeric anthocyanin concentration (TAC) was determined using a spectrophotometric pH differential method [29]. The TAC is expressed as milligrams (mg) C3G equivalent with a molar extinction coefficient 26,900 and a molecular weight of 449.2 g/mol. C3G, PGA, and PCA were quantified as described before by [28] using ultra HPLC electrospray ionization tandem mass spectrometry (UPLC-ESI-MS/MS) on a Waters H-class UPLC separations module (Waters, Milford, MA, USA) coupled with a Micromass Quattro micro API MS/MS system and MassLynx V4.0 control software (Micromass, Cary, NC, USA). The column used was an Aquity BEH C18 (100 mm × 2.1 mm × 1.7 µm) (Waters, Milford, MA, USA). PCA and PGA were analyzed using single ion monitoring mode (SIM) with electrospray ionization in negative mode (ESI−) with a capillary voltage of 3000 V, a nebulizer gas temperature (N_2_) of 375 °C, and a flow rate of 0.35 mL/min. C3G was analyzed using electrospray ionization in positive ion mode (ESI+), with a capillary voltage of 3500 V, a nebulizer gas temperature (N_2_) of 375 °C, and a flow rate of 0.35 mL/min. Samples were diluted 1:1 with methanol, then filtered through 0.22 μm nylon filters into amber glass UPLC vials.

#### 2.4.3. Statistical Analysis

Repeated measures analysis (*p* ≤ 0.05) was performed using the statistical analysis system software (SAS Institute, Cary, NC, USA). The data for C3G incubated at 90 °C was normalized using a log_10_ transformation.

### 2.5. Antiproliferative Activity

#### 2.5.1. Preparation of Extracts

PE was prepared for cell-based assays by dissolving freeze-dried PE to a concentration of 60 mg/mL in deionized water (DI H_2_O). Degraded extracts were prepared by subjecting aliquots of this PE solution to thermal degradation at 90 °C in a shaking water bath for 2 h (HPE2) or 8 h (HPE8). The crude extract (CE) was prepared by dissolving freeze-dried CE to a concentration of 60 mg/mL in DI H_2_O.

#### 2.5.2. Cell Culture

Breast adenocarcinoma (MDA-MB-231; American Type Culture Collection (ATCC) # HTB 26) cells were obtained from the American Type Culture Collection (ATCC, Manassas, VA, USA) and cultured at 37 °C with 5% CO_2_, in DMEM with 2 mM l-glutamine, 10% heat-inactivated FBS, 1% 4-(2-hydroxyethyl)-1-piperazineethanesulfonic acid (HEPES), and 1% penicillin–streptomycin. Hepatocellular carcinoma (HepG2; ATCC # 8065) cells were also obtained from the ATCC and cultured in EMEM with 2 mM l-glutamine, 10% FBS, and 1% penicillin-streptomycin at 37 °C with 5% CO_2_.

#### 2.5.3. Cell Viability MTS Assay

A CellTiter 96^®^ AQ_ueous_ One Solution Cell Proliferation Assay (Promega, Madison, WI, USA) was used to determine cell viability. Cells were seeded in 96-well plates at a density of 5 × 10^3^ cells per well and incubated for 24 h to promote cell adhesion. Cells were treated with PE, HPE2, HPE8, or CE. For HepG2 cells, treatments were added at concentrations of 75, 150, and 300 µg/mL with a positive control of sorafenib in DMSO with a final concentration of 10 µg/mL and negative controls consisting of either EMEM with: 0.5% DI H_2_O for PE, HPE2, HPE8, or CE treatments, or 0.5% DMSO for PCA or PGA treatments. For MDA-MB-231 cells, treatments were added at concentrations of 50, 100, and 200 µg/mL with a positive control of doxorubicin in DMSO at a final concentration of 50 µM, and negative controls of DMEM with either 0.5% DI H_2_O (PE, HPE2, HPE8, or CE) or 0.5% DMSO (PCA, PGA). After either a 24 or 48 h incubation, 20 µL of MTS was added to each well. Plates were incubated for three hours at 37 °C in a 5% CO_2_ atmosphere before absorbance was measured at 490 nm using a FLUOstar Optima microplate reader (BMG Labtech, Ortenberg, Germany). Cell viability calculated with treated cells being expressed as a percent of cells treated with vehicle only.

#### 2.5.4. Acid Phosphatase Assay

Acid phosphatase buffer was prepared with 0.1 M sodium acetate (NaAc), 0.1% *v*/*v* Triton X-100, and 4 mg/mL phosphatase substrate (Sigma-Aldrich, Oakville, ON, Canada), adjusted to a pH of 5.5 with HCl, and stored at 4 °C. HepG2 cells were treated with PE, PCA, and PGA at concentrations of 75, 150, and 300 µg/mL with a positive control of sorafenib in DMSO with a final concentration of 10 µg/mL, and negative controls consisting of EMEM with either: 0.5% DI H_2_O for PE treatments, or 0.5% DMSO for PCA or PGA treatments. After incubation for a period of 48 h, plates were centrifuged at 2000 rpm for 10 min before the supernatant was removed and 100 µL of PBS added to each well. Plates were again centrifuged before PBS was removed and 100 µL of assay buffer was added to each well. Plates were then incubated for two hours at 37 °C in a 5% CO_2_ atmosphere. After incubation, 10 µL of 1 N sodium hydroxide was added to each well, and absorbance was measured at 405 nm using a FLUOstar Optima microplate reader (BMG Labtech, Ortenberg, Germany). Cell viability was calculated as described in Section 2.5.3.

#### 2.5.5. Adenosine Triphosphate (ATP) Assay

The CellTiter-Glo^®^ Luminescent Cell Viability Assay (Promega, Madison, WI, USA) was used to determine cell viability and ATP levels. HepG2 cells were treated under the same conditions as those in the acid phosphatase assay. The CellTiter-Glo^®^ reagent was prepared as directed by the manufacturer from CellTiter-Glo^®^ substrate and the CellTiter-Glo^®^ buffer, and 100 µL of reagent was added to each well. Plates were shaken for 2 min at 60 rpm using a VWR orbital shaker (Troemner LLC, Mini Shaker, Thorofare, NJ, USA) to induce cell lysis followed by a 10-min room-temperature incubation. Luminescence was measured using a FLUOstar Optima microplate reader (BMG Labtech, Ortenberg, Germany). Cell viability was calculated as described in Section 2.5.3.

#### 2.5.6. Statistical Analysis

All experiments were designed using completely randomized design. Assays were performed in triplicate (*n* = 3), and all results were expressed as the mean ± standard deviation (SD). Analyses were compared using one-way analysis of variance (ANOVA) and Tukey’s test (*p* ≤ 0.05). Statistical analyses were carried out using the Minitab v.17 software (Minitab, State College, PA, USA) package.

## 3. Results

### 3.1. C3G Degradation

**Figure 1:**
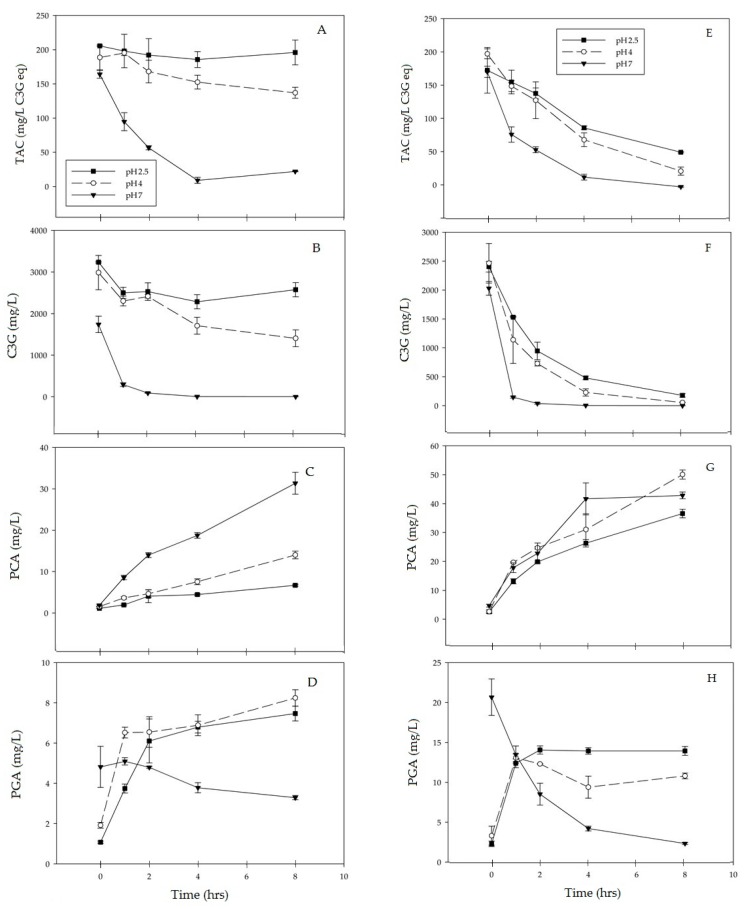
Total monomeric anthocyanin (TAC), cyanidin-3-*O*-glucoside (C3G), protocatechuic acid (PCA) and phloroglucinaldehyde (PGA) content at 70 °C (**A**–**D**) and 90 °C (**E**–**H**). TAC was measured using pH differential spectrophotometric assay and quantification of C3G, PCA, and PGA by ultra HPLC electrospray ionization tandem mass spectrometry (UPLC-ESI-MS/MS).

Monomeric anthocyanins’ degradation was more pronounced in extracts incubated at 90 °C than at 70 °C (Figure 1A,E). Also, extracts incubated at the pH of 7.0 exhibited significantly greater anthocyanin degradation than extracts incubated in acidic pH conditions. As demonstrated in Figure 1B,F, at incubation temperatures of 70 °C and 90 °C, the breakdown of C3G in haskap PE was significantly affected (*p* ≤ 0.05) by both incubation time and pH.

As the pH increased, C3G degraded more readily, with extracts incubated at 90 °C degrading more quickly than those incubated at 70 °C. Extracts incubated for 8 h at pH 2.5, lost 21% of the initial C3G content at 70 °C, and lost 95% of the initial C3G content at 90 °C. Extracts at pH 4.0 followed a similar trend losing 53% of the C3G content at 70 °C versus a 98% loss at 90 °C. At pH 7.0, C3G was almost completely lost at both 70 °C and 90 °C. C3G is the least stable at pH 7 when compared to pH 2.5 and pH 4.0 (Figure 1B,F).

### 3.2. Formation of PCA

The accumulation of PCA, one of C3G’s major breakdown products, was quantified in the thermally degraded purified extracts. PCA concentration increased as C3G degraded under all tested conditions. Time and pH both significantly (*p* ≤ 0.05) affected the accumulation of PCA as C3G degraded. The highest concentration of accumulated PCA, 50.0 mg/L, was observed over an 8-h incubation at pH 4 and 90 °C. As with C3G degradation, at 70 °C the largest differences in PCA concentrations were found when comparing pH 7.0 to an acidic pH of 2.5 or 4.0. At 90 °C variations were less dramatic, suggesting that the presence of PCA in C3G-rich extracts is an indicator of C3G degradation (Figure 1C,G).

### 3.3. Formation of PGA

PGA concentration (Figure 1D,H) showed less of an association to C3G degradation than PCA concentration. At 70 °C and 90 °C, and pHs of 2.5 and 4.0, extracts showed an increase in PGA concentration, with most of the accumulation occurring during the first 2 h of the 8-h incubation. Depending on incubation conditions, after the initial 2 h, PGA accumulation decelerated, stabilized, or for pH 7.0 at both 70 °C and 90 °C PGA concentration decreased.

**Figure 2:**
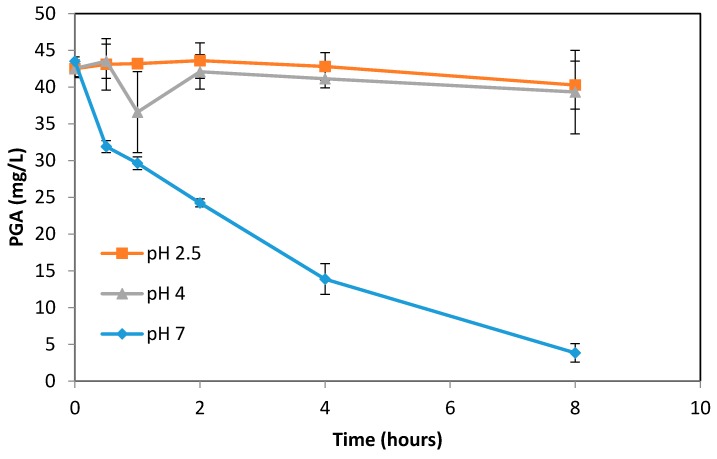
Phloroglucinaldehyde (PGA) concentration at 90° C at pH 2.5, 4, and 7, respectively.

To understand whether the PGA can be further degraded at pH 7 after 2 h, the second experiment was performed using pure PGA (Figure 2). PGA heated at pH of 2.5 and 4 showed minimal degradation over the 8 h period. However, at pH 7, the PGA experienced rapid degradation and exhibited over 90% loss after 8 h of heating. This is in agreement with the previous experiment, which found that PGA accumulated from C3G degradation had decreased concentrations at pH 7. A visible color change could be seen with PGA solutions mixed with the pH 7 buffer; PGA solutions at pH 2.5 and 4 remained colorless, while pH 7 solutions became increasingly yellow with longer heating times.

### 3.4. Breast Adenocarcinoma MDA-MB-231 Viability

The viability of treated MDA-MB-231 cells was assessed using an MTS assay. PE, HPE2, HPE8, CE, PCA, and PGA were used to treat the cells. The concentrations of C3G, PCA, and PGA in each extract are displayed in Table 1. Cellular viability was measured by comparing cells treated with extracts and commercially purchased PCA and PGA, at concentrations of 50, 100, and 200 μg/mL, to negative and positive controls. The negative control consisted of DMEM growth medium with 0.5% DMSO, and the positive control was 0.5% DMSO with the chemotherapy drug doxorubicin at a concentration of 50 μM.

None of the haskap-derived treatments affected cellular viability except PGA (Table 2). At concentrations of 100 µg/mL and above, PGA significantly reduced MDA-MB-231 viability over periods of 24 and 48 h. In both cases, a cell viability of 35% was observed; with a strong dose-dependent trend but no clear time-dependence.

### 3.5. Hepatocellular Carcinoma HepG2 Cell Viability

The viability of HepG2 cells, after exposure to haskap berry extracts containing C3G and its metabolites, was assessed using an MTS assay, an acid phosphatase assay, and an ATP assay. Cellular viability was compared to a negative control consisting of EMEM growth medium with 0.5% DMSO, and a positive control containing 10 μg/mL of the chemotherapy drug sorafenib. Cells were treated with PE, HPE2, HPE8, CE, PCA, and PGA at concentrations of 75, 150, and 300 μg/mL.

MTS-based viability determination (Table 2) showed a dose- and time-dependent reduction in cell viability for PE and PGA. After a 24-h incubation, cell viability was 52% with a PGA concentration of 75 μg/mL, and only 20.2% with a concentration of 300 μg/mL, when compared to the negative control. For PE at 300 μg/mL after a 24-h incubation, cell viability was 64% compared to the negative control. PCA treatments showed no effect on HepG2 viability as determined via MTS assay. Treatments showing dose-dependent inhibition (PE & PGA), along with PCA due to conflicting reports of its anti-tumorigenic activity, were selected for further testing over 48-h incubations.

**Figure 3:**
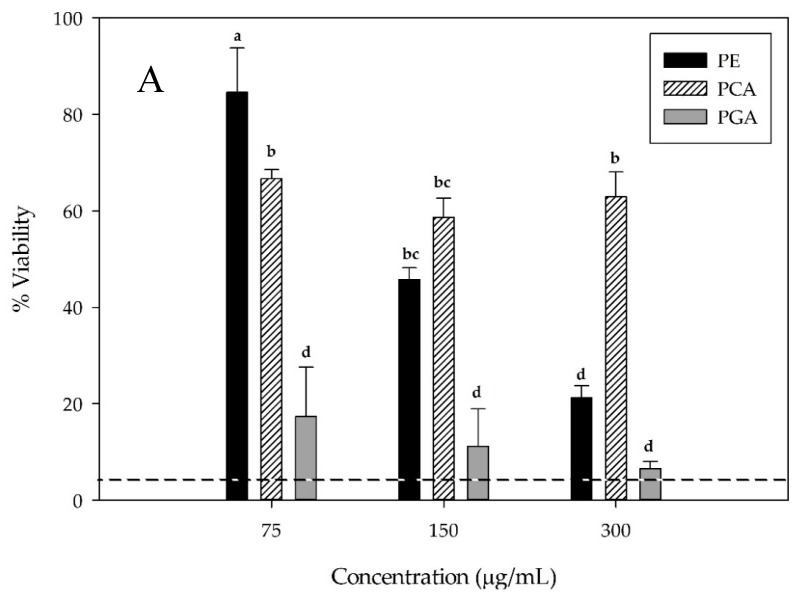

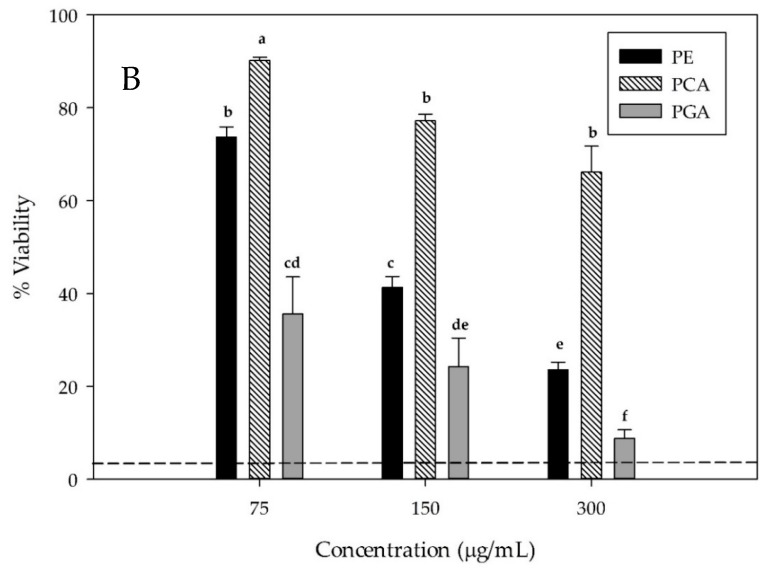
Cellular viability (% relative to the control) as determined by an acid phosphatase assay (**A**) and ATP content (**B**) of HepG2 cells incubated with C3G-rich fraction (PE), PCA, and PGA for 48 h. Dashed line represents positive control sorafenib at a concentration of 10 µg/mL. Letters (a–f) on the bars indicate significantly different means (*p* ≤ 0.05). PE, C3G-rich fraction; PCA, protocatechuic acid; PGA, phloroglucinaldehyde.

Cell viability, as determined in an acid phosphatase assay, showed that PE (Figure 3A) induced a strong dose-dependent reduction in cellular viability. This reduction ranged from 84.5% at a dosage of 75 μg/mL, to 21.2% at 300 μg/mL, as seen in Figure 3A. PGA also induced a significant reduction in cell viability, with all three treatments exhibiting similar activity to the positive control. PCA showed no significant dose-dependent reduction in cellular viability.

It was determined via an ATP assay that PE-induced a dose-dependent reduction in ATP activity after an incubation of 48 h (Figure 3B). PE added at a concentration of 75 μg/mL did not generate a value that was significantly different from the vehicle control (*p* ≤ 0.05); however, PE added at a concentration of 300 μg/mL induced an ATP reduction to 23.5%. PGA induced a strong reduction in detectable ATP, ranging from 35.6% of luminescence at a concentration of 75 μg/mL, to 8.8% at a concentration of 300 μg/mL (Figure3B). PCA induced a slight dose-dependent reduction in luminescence with 150 μg/mL and 300 μg/mL being significantly different than the negative control, but not significantly different from each other.

## 4. Discussion

Numerous studies and reviews have investigated and confirmed that anthocyanins undergo first-order kinetic degradation [30,31,32,33,34]. At a neutral pH, anthocyanins are liable to undergo spontaneous degradation, making the treatment temperature less significant for samples at neutral pH [16,30,35]. As demonstrated in Figure 1 and Figure 2, for samples degraded at both 70 °C and 90 °C at time 0, samples at pH 7.0 showed a C3G reduction of almost 50% compared to the samples at pH 2.5. Within a given pH, C3G degradation accelerates at higher temperatures. This indicates that for C3G containing products, lower-temperature processing may preserve anthocyanin content compared to higher temperature methods. A study by Khattab et al. [36] monitored anthocyanin degradation in the drying of whole haskap berries at temperatures of 60, 100, and 140 °C. The study found that reductions of 74–76%, 79–81%, and 91–95%, respectively, of total anthocyanins were observed at those temperatures. Among the five predominant anthocyanins present in haskap berries, C3G was the least thermally stable [36].

Along with affecting the rate of C3G degradation, solution pH may also affect the mechanism of degradation. For C3G at a pH of 3.5, it appears that the opening of the pyrylium ring initiates anthocyanin degradation, forming a glycosylated chalcone [37]. For C3G at a pH of 1, deglycosylation was proposed as the first step in the degradation pathway [20]. It has been determined that the addition of sugars could help prevent anthocyanin degradation under some storage and treatment conditions [38]. This could be valuable, especially at higher temperatures and both acidic and neutral pH.

As with C3G degradation, the widest variation in PCA concentration was observed between measurements taken at pH 2.5 and 7.0 at 70 °C, with there being slightly less variation at 90 °C. This could indicate an inverse relationship, where PCA concentration could potentially represent the level of C3G degradation (Figure 1). The value of PCA as a bioactive compound is still unknown and PCA does not impart vibrant color, so its value for commercial products is still undetermined. Further investigation into the bioactivity of PCA and its interactions is recommended.

For incubation times longer than 2 h, at both 70 °C and 90 °C, PGA accumulation slowed or stabilized. For all samples at pH 7.0, there was a decrease in PGA concentration over time. The decelerating of PGA accumulation could be attributed to PGA degradation. Incubation of pure PGA at 90 °C at pH 7.0 showed its further degradation (Figure 2). This indicates that, like its parent anthocyanin C3G, PGA is unstable at higher pH, and further degrades as the incubation time increases. High pH values appear to have a prominent effect on the degradation of PGA, which is further accelerated by heating.

Haskap extract did not significantly reduce MDA-MB-231 cell viability. This is likely due to differing anthocyanin profiles between haskap berries and blueberries. Interestingly, PGA significantly reduced the MDA-MB-231 viability, which has not previously been reported. PGA has been shown to be anti-proliferative when incubated with Caco-2 cells in vitro [24,25]. Further studies should be conducted to determine if this reduction in viability is selective, and to elucidate the underlying mechanism. For HepG2 cells exposed to haskap berry extracts, and C3G metabolites, CE reduced cell viability to 64% when treated at a level of 300 μg/mL. Based on the ATP assay, PCA caused a significant reduction in HepG2 viability. PCA has been reported to reduce HepG2 viability [39]. The results of the MTS, ATP, and acid phosphatase assays suggest that PCA and PGA in thermally degraded haskap extracts may not be present in high enough concentrations to induce cytotoxicity in both cancer cell lines. However, both PE and PGA caused dose-dependent inhibition of HepG2.

## 5. Conclusions

Solution pH, incubation time, and temperature affect anthocyanin stability. As these increase, the anthocyanin concentration decreases. C3G is more stable when treated at lower temperatures over longer incubations when compared to shorter incubations at higher temperatures. Incubation at pH 7.0 promotes rapid C3G degradation; therefore, maintaining an acidic pH during processing is favorable for C3G retention. Haskap juice is naturally acidic in nature, and this acidity should be preserved to maintain anthocyanin stability. Haskap berry extract with high levels of C3G produced a time- and dose-dependent inhibition of HepG2 cell proliferation and PGA generated dose-dependent inhibition of MDA-MB-231 cells. This preliminary in vitro study suggests that haskap berry anthocyanins could potentially have cancer chemopreventive properties. It also indicates that the inhibitory effects of haskap berry extracts are mediated, at least in part, by C3G. Further studies are required to determine the anti-cancer potential and mechanism of action of C3G. One of C3G’s thermal degradation product and in vivo primary metabolites, PGA, also generated significant anti-proliferative effects on HepG2 cells. Processing of haskap berries and their storage could exacerbate anthocyanin degradation, which could lead to a reduction in putative health benefits for the consumer. Since there is growing interest in the use of fruit-derived C3G as a food additive, natural colorant, dietary antioxidant, and nutraceutical, further research is required to determine the toxicity and biological activity of PGA.

## Figures and Tables

**Table 1:** The composition of the C3G-rich extracts and thermally-challenged extracts (500 µg/mL) used for the cell viability analyses.

Treatment	C3G (µg/mL)	PCA (µg/mL)	PGA (µg/mL)
PE	59.92 ± 1.6	0.28 ± 0.001	0.023 ± 0.0002
HPE2	34.23 ± 3.3	0.77 ± 0.04	0.29 ± 0.01
HPE8	5.76 ± 0.5	2.02 ± 0.2	0.467 ± 0.01
CE	11.35 ± 0.1	N/A	N/A

C3G, Cyanidin-3*-O*-glucoside; PCA, protocatechuic acid; PGA, phloroglucinaldehyde; PE, C3G-rich fraction; HPE2, the PE subjected to 90 °C for 2 h; HPE8, the PE subjected to 90 °C for 8 h; CE, crude extract.

**Table 2:** Cell viability measured using the MTS assay.

Treatment	Concentration	24 h	48 h
	(µg/mL)	(% Viability ± SD)	(% Viability ± SD)
**MDA-MB-231**			
PE	50	97.43 ± 2.04 ^ab^	103.10 ± 10.17 ^a^
	100	107.02 ± 6.71 ^ab^	105.15 ± 5.29 ^a^
	200	109.86 ± 1.50 ^ab^	106.52 ± 6.50 ^a^
HPE2	50	100.22 ± 2.14 ^cd^	98.34 ± 0.91 ^a^
	100	104.96 ± 1.42 ^ab^	106.43 ± 4.20 ^a^
	200	108.38 ± 4.16 ^ab^	94.13 ± 8.44 ^a^
HPE8	50	103.82 ± 2.48 ^ab^	107.68 ± 6.24 ^a^
	100	104.19 ± 4.13 ^ab^	104.78 ± 4.45 ^a^
	200	100.55 ± 3.11 ^ab^	85.10 ± 11.13 ^a^
CE	50	103.31 ± 4.98 ^ab^	105.37 ± 3.46 ^a^
	100	99.33 ± 3.24 ^ab^	98.99 ± 12.10 ^a^
	200	98.73 ± 2.18 ^ab^	99.16 ± 3.86 ^a^
PCA	50	101.18 ± 11.40 ^ab^	94.43 ± 6.65 ^a^
	100	101.08 ± 8.12 ^ab^	81.96 ± 13.33 ^a^
	200	107.89 ± 10.21 ^ab^	86.96 ± 29.52 ^a^
PGA	50	78.22 ± 2.37 ^bc^	80.35 ± 5.54 ^a^
	100	35.30 ± 26.66 ^d^	36.25 ± 5.11 ^bc^
	200	7.09 ± 8.93 ^e^	17.71 ± 13.55 ^c^
Dox	27.2	53.30 ± 13.56 ^cd^	7.89 ± 7.29 ^c^
**HepG2**			
PE	75	89.26 ± 11.01 ^bc^	72.9 ± 3.07 cd
	150	88.39 ± 2.43 ^bc^	70.97 ± 7.32 d
	300	64.32 ± 14.33 ^cd^	35.57 ± 11.55 e
HPE2	75	105.92 ± 7.78 ^ab^	92.09 ± 4.61 ^abc^
	150	104.28 ± 9.19 ^ab^	91.26 ± 5.18 ^abcd^
	300	97.34 ± 8.48 ^ab^	81.58 ± 6.69 ^bcd^
HPE8	75	105.64 ± 8.35 ^ab^	90.15 ± 7.96 ^abcd^
	150	102.85 ± 5.86 ^ab^	81.65 ± 10.15 ^bcd^
	300	103.35 ± 5.08 ^ab^	80.07 ± 6.22 ^bcd^
CE	75	102.92 ± 3.59 ^ab^	95.2 ± 5.03 ^ab^
	150	102.59 ± 7.62 ^ab^	94.67 ± 3.6 ^ab^
	300	104.76 ± 4.90 ^ab^	93.35 ± 3.29 ^abc^
PCA	75	84.98 ± 4.04 ^bc^	90.943 ± 1.6 ^abcd^
	150	92.41 ± 8.27 ^ab^	89.99 ± 7.21 ^abcd^
	300	114.06 ± 7.53 ^ab^	108.66 ± 7.77 ^a^
PGA	75	52.85 ± 3.82 ^de^	25.71 ± 7.07 ^ef^
	150	18.34 ± 10.46 ^f^	15.16 ± 7.36 ^efg^
	300	20.23 ± 8.25 ^f^	1.80 ± 1.70 ^g^
Sorafenib	10	39.38 ± 5.48 ^ef^	11.40 ± 9.22 ^fg^

Results represent the mean ± SD, Letters (a–g) indicate significantly different means (*p* ≤ 0.05) within temperature treatments. MTS: 5-[3-(Carboxymethoxy) phenyl]-3-(4,5-dimethyl-2-thiazolyl)-2-(4-sulfophenyl)-2H-tetrazolium inner salt.
